# Supplementary material for: Mahan excitons in room-temperature methylammonium lead bromide perovskites
Source: Nat Commun. 2020 Feb 12;11:850. doi: 10.1038/s41467-020-14683-5 (PMC7016123; doi:10.1038/s41467-020-14683-5)
Supplement: Supplementary file 1 — Supplementary Information [file 41467_2020_14683_MOESM1_ESM.pdf]

Supplementary Information

Mahan excitons in room-temperature methylammonium lead bromide perovskites

Palmieri *et al.*

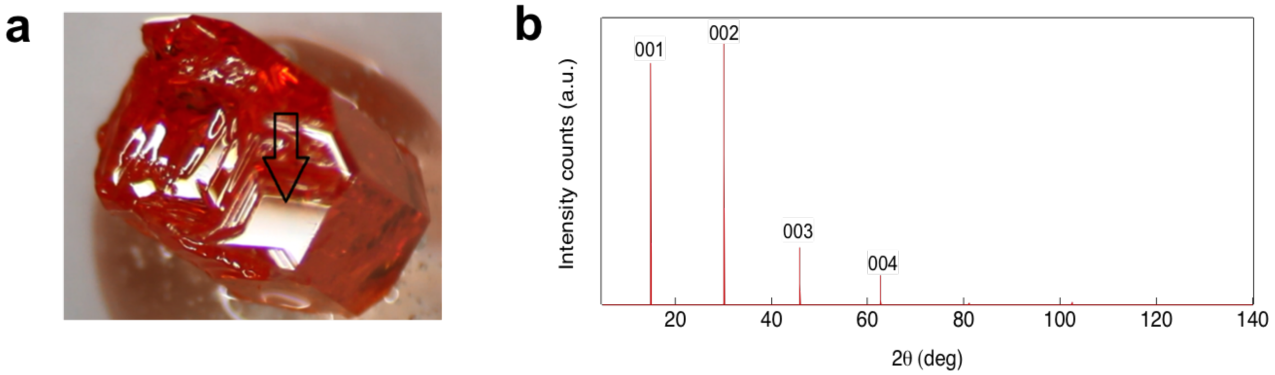

Supplementary Figure 1: (**a**) Surface cleaved from a cluster of intergrown $CH_3NH_3PbBr_3$. (**b**) X-ray diffraction pattern of $CH_3NH_3PbBr_3$ single crystals, showing sharp peaks and the absence of any degradation.

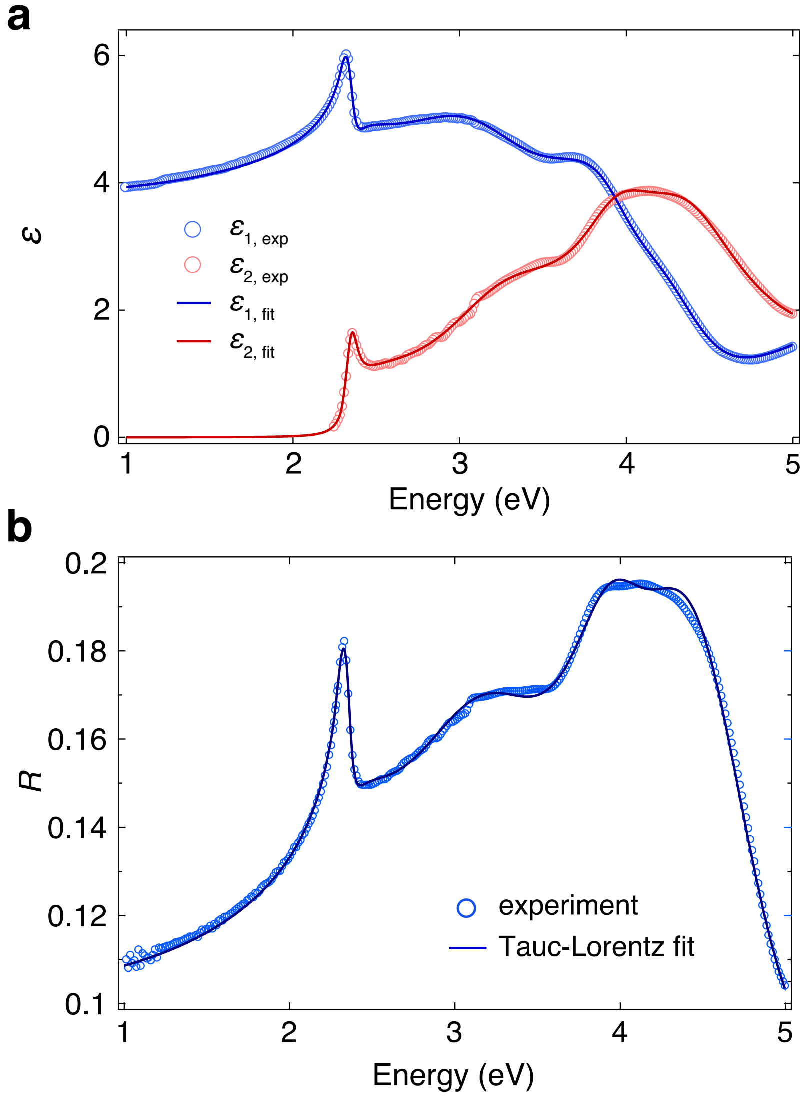

Supplementary Figure 2: (**a**) Real (ε_1, blue) and imaginary (ε_2, red) parts of the dielectric function $\varepsilon = \varepsilon_1 + i\varepsilon_2$ of $CH_3NH_3PbBr_3$ single crystals measured by spectroscopic ellipsometry. The experimental data (dots) are fitted with a Tauc-Lorentz model (solid line). (**b**) Reflectivity spectrum of $CH_3NH_3PbBr_3$ single crystals calculated from the ellipsometry data (dots) overlapped with the fit obtained via Tauc-Lorentz model (solid line).

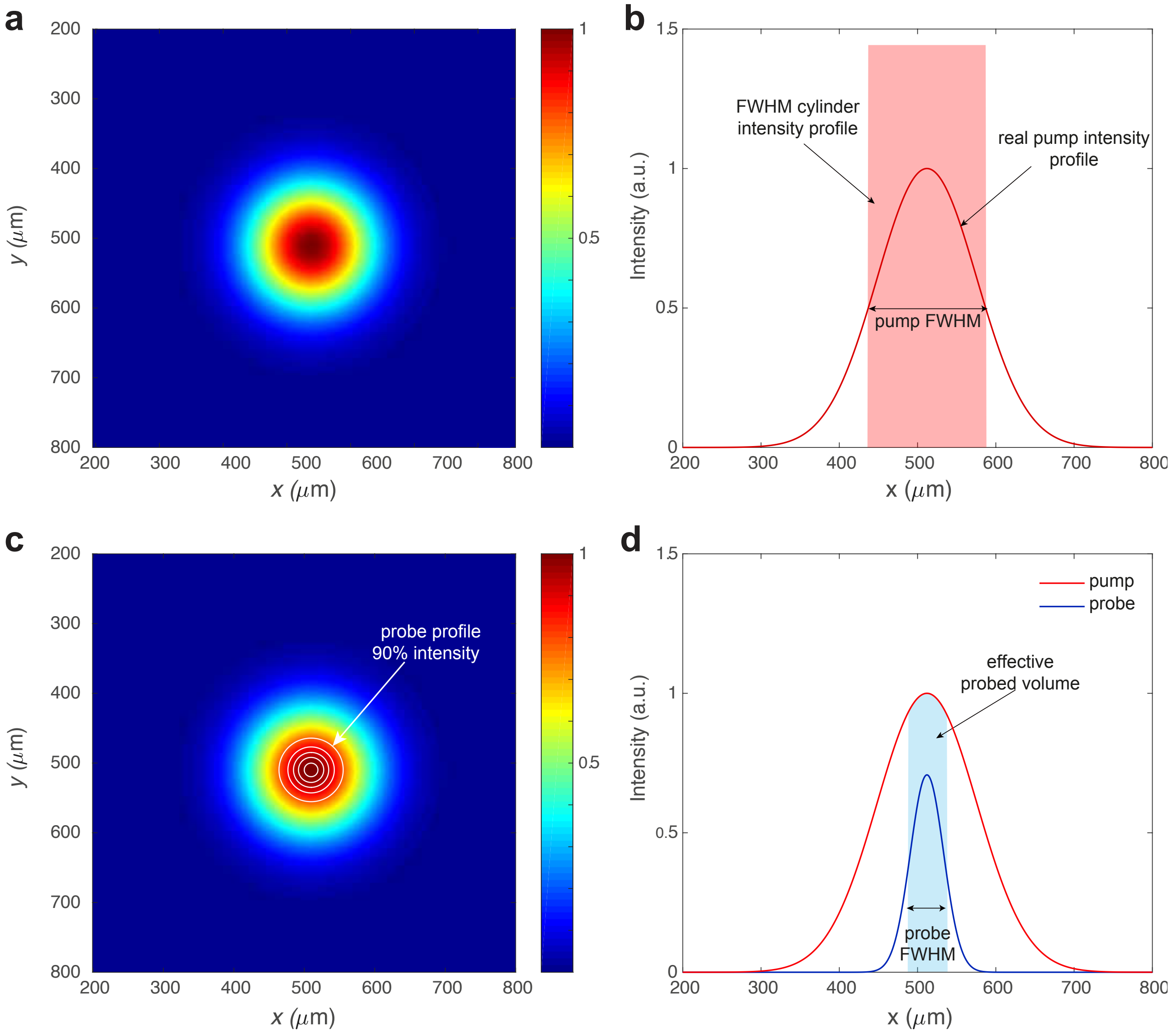

Supplementary Figure 3: (**a**) Simulation of the gaussian excitation beam used in our experiment (FWHM = 150μm). (**b**) Profiles of the two-dimensional intensity distributions of the gaussian beam of panel (a) (red curve) and the one obtained by approximation of the gaussian beam with a cylinder (red rectangule). (**c**) Simulated gaussian intensity distributions of the pump (colour-coded) and probe (white contour lines) beams used in the experiment. (**d**) Profiles of the two-dimensional intensity distributions of the pump and probe beams of panel (c), showing that in these experimental conditions the probed area coincides with the most intense part of the gaussian.

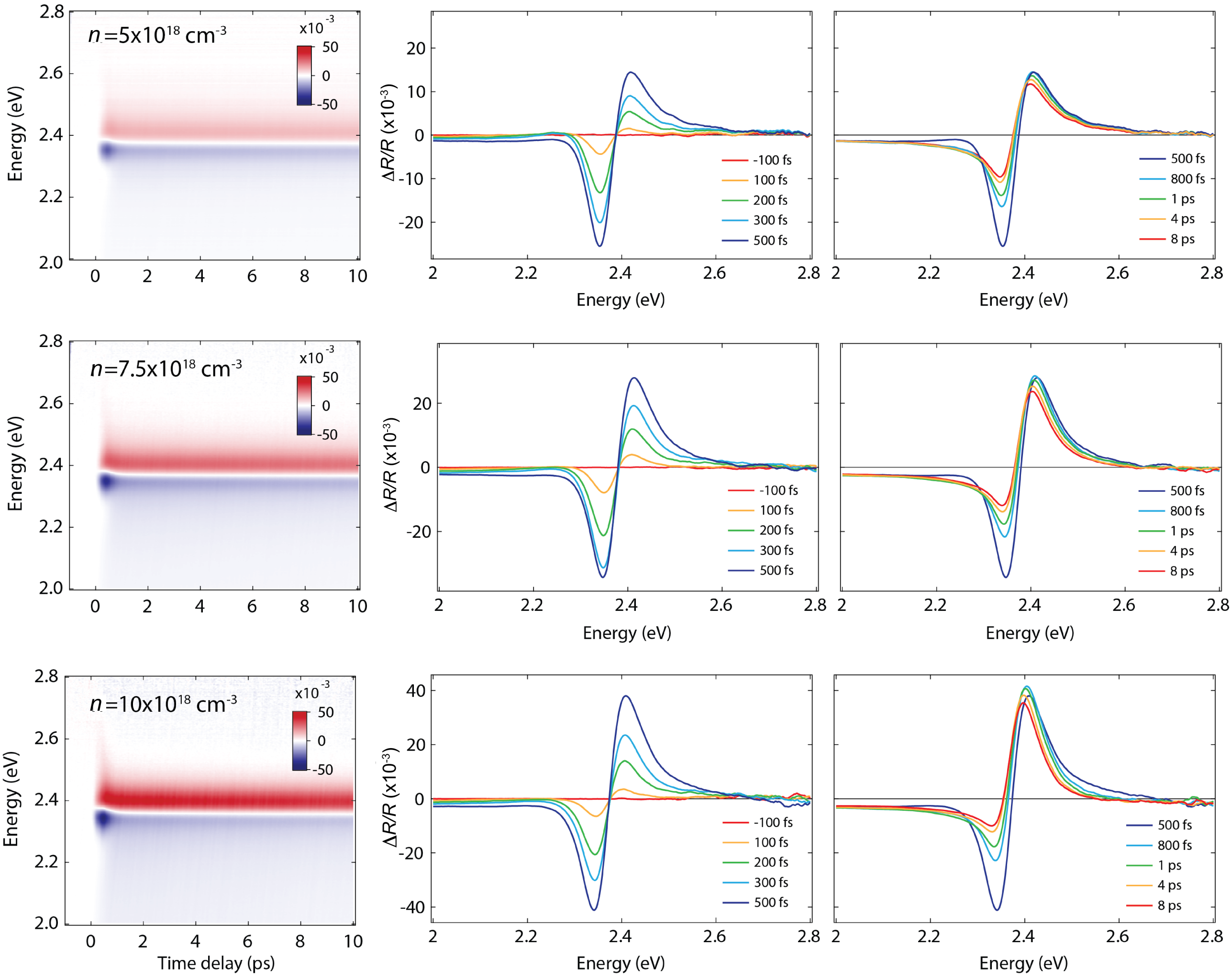

Supplementary Figure 4: Colour-coded $\Delta R/R$ maps for the pump excitation densities of 5, 7.5 and 10×10^{18} cm^{-3} and corresponding transient spectra from -100 fs to 500 fs and from 500 fs to 8 ps.

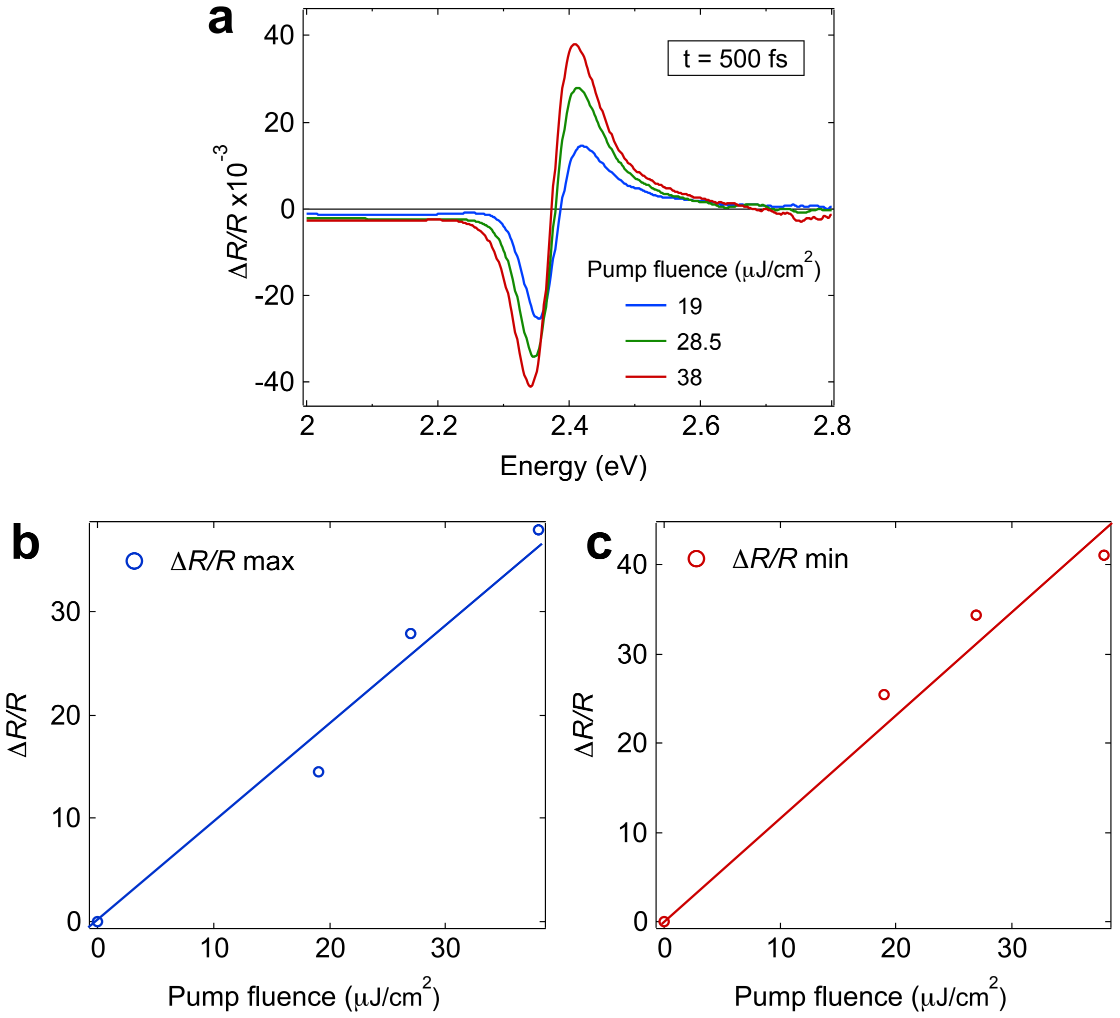

Supplementary Figure 5: (**a**) Comparison between the $\Delta R/R$ in the range 19–38 μJ/cm^2 at 500 fs (*i.e.* the time at which the response amplitude is maximum), showing no evidence of signal saturation. (**b,c**) Evolution of the maximum (b) and minimum (c) $\Delta R/R$ signal (absolute value) with increasing fluence.

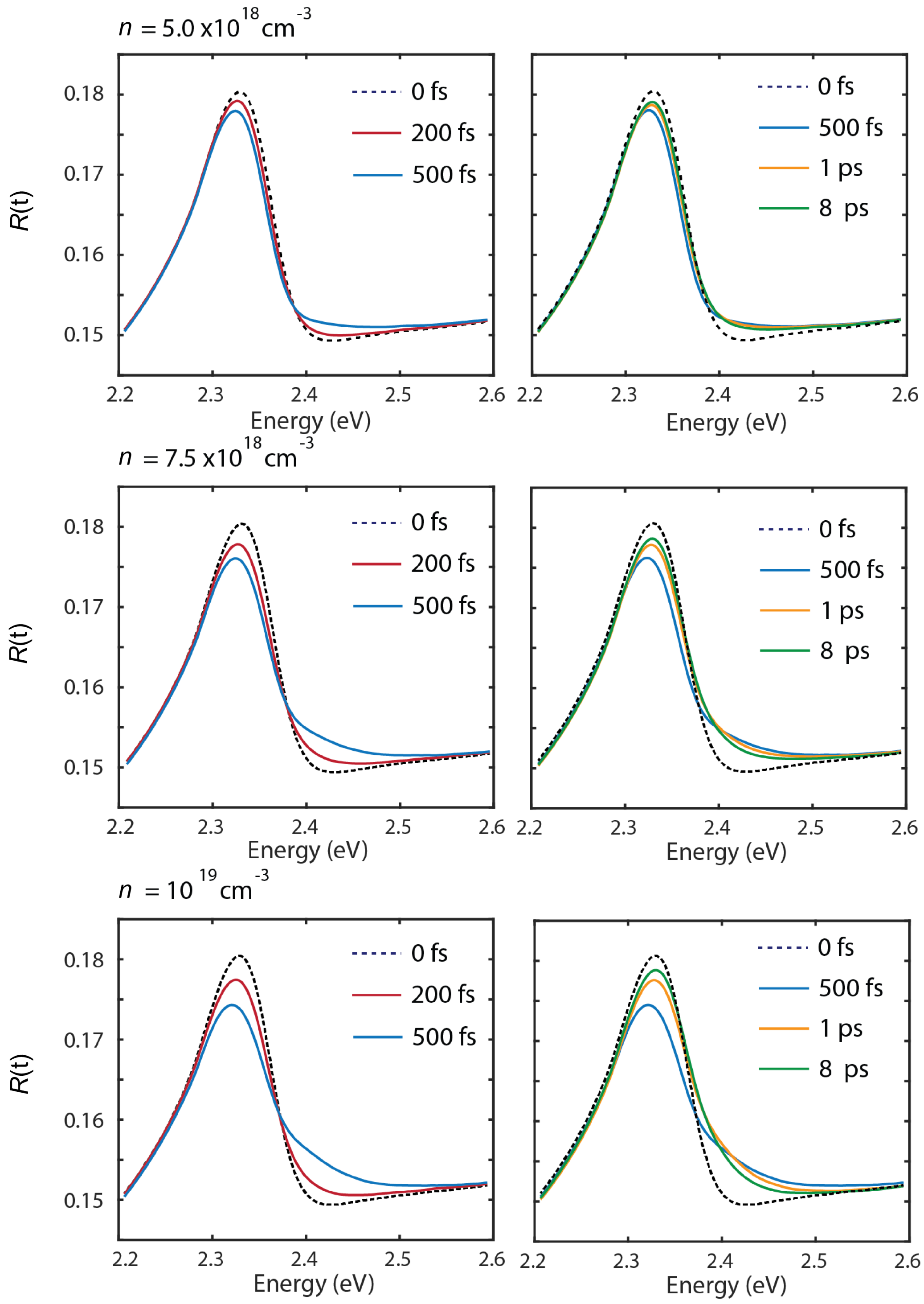

Supplementary Figure 6: Spectral evolution of $R(\omega, t)$, obtained by combining the steady-state $R_s(\omega)$ calculated from ellipsometry and the $\Delta R/R(\omega, t)$ data from 0 to 500 fs and from 500 fs to 8 ps. The excitation density is indicated on top of each panel row.

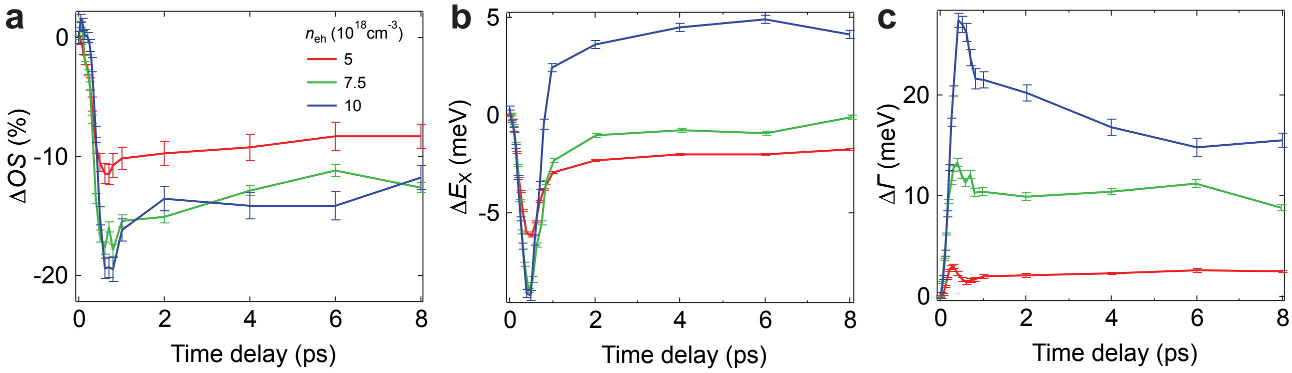

Supplementary Figure 7: Temporal evolution of (**a**) the peak position (ΔE_{X}), (**b**) oscillator strength (ΔOS), and (**c**) linewidth ($\Delta \Gamma$) with respect to the equilibrium parameters as obtained through the Tauc-Lorentz fit of $\Delta R/R(\omega, t)$ at the excitation density of $n = 5, 7.5$ and 10×10^{18}cm^{-3}.

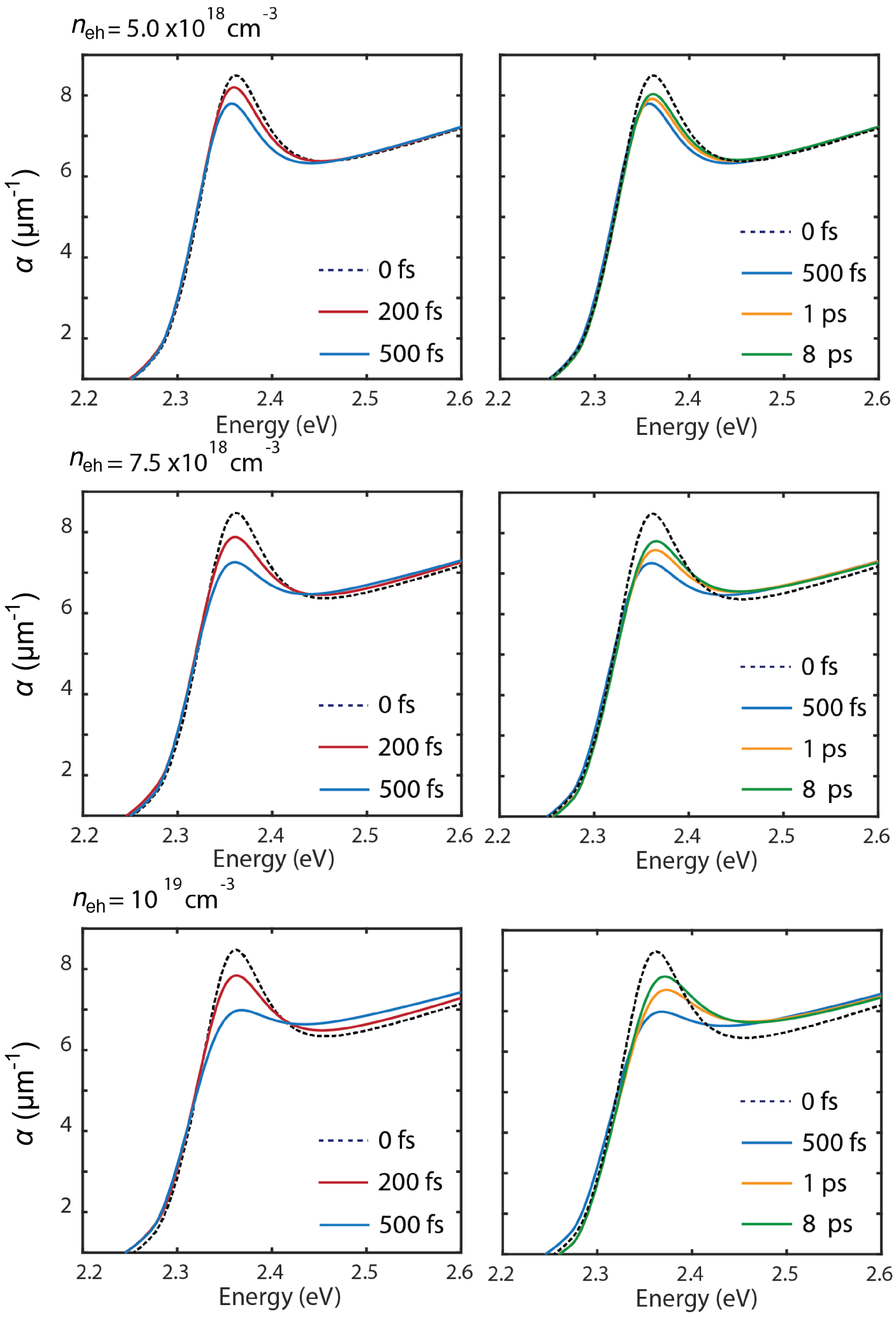

Supplementary Figure 8: Time-resolved absorption spectra calculated from the evolution of the TL parameters from 0 to 500 fs and from 500 fs 8 ps. The excitation density is indicated on top of each panel row.

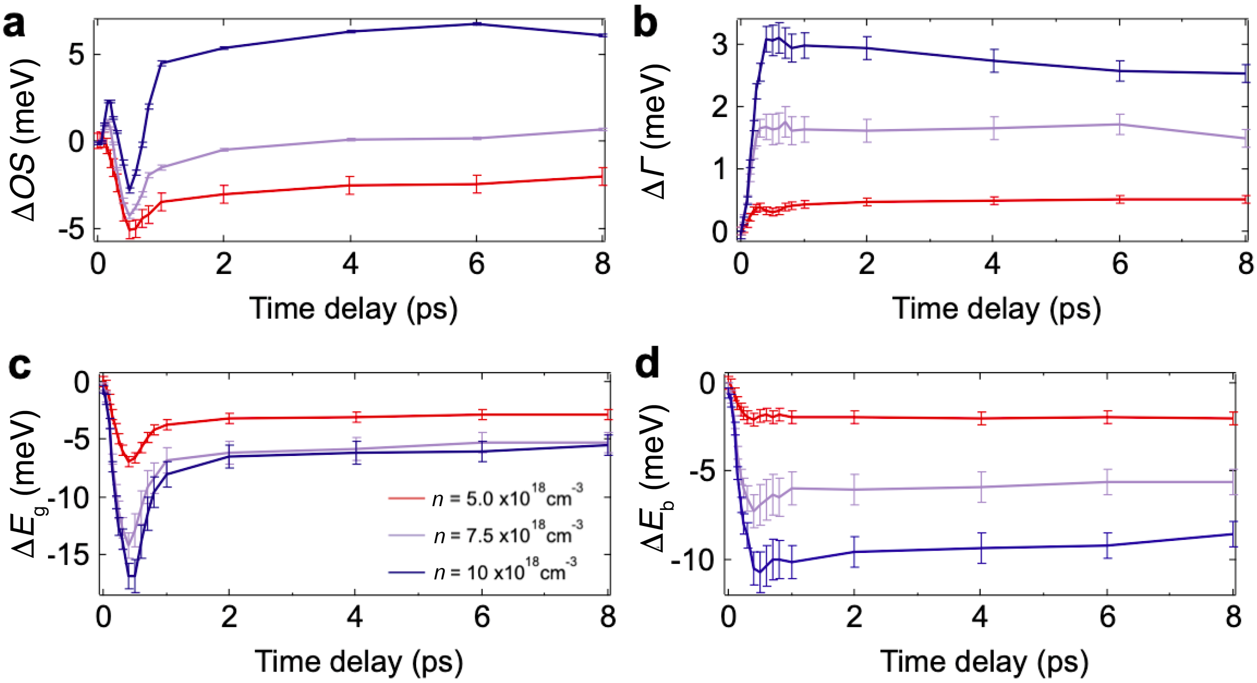

Supplementary Figure 9: Evolution of (**a**) oscillator strength (ΔOS), (**b**) linewidth ($\Delta \Gamma$), (**c**) bandgap energy (ΔE_{g}), and (**d**) exciton binding energy (ΔE_{b}) with respect to the equilibrium exciton parameters. The curves are obtained by iterative fit of the absorption at different time delays with Elliott model.

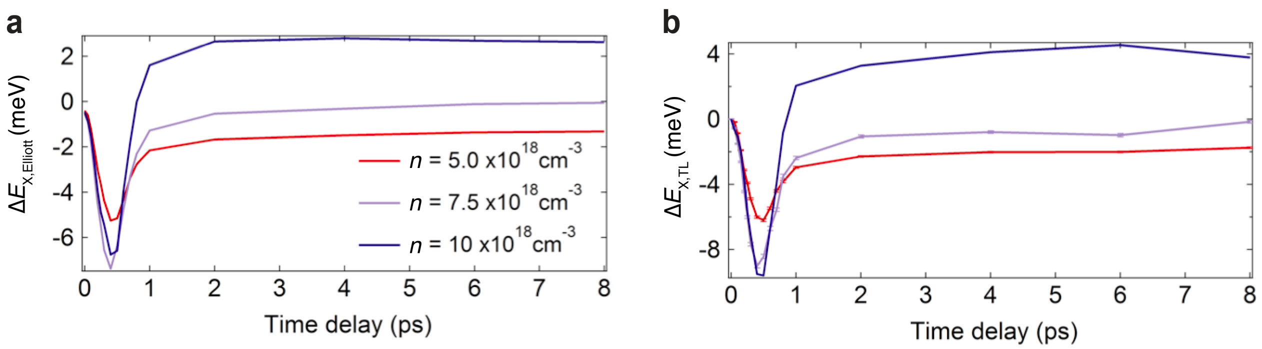

Supplementary Figure 10: Evolution of the exciton peak position as obtained from the iterative fit of $\alpha(\omega, t)$ with Elliott model ($\Delta E_{\text{X, Elliott}}$, (**a**)) and from the iterative fit of $R(\omega, t)$ with Tauc-Lorentz model ($\Delta E_{\text{X, TL}}$, (**b**)).

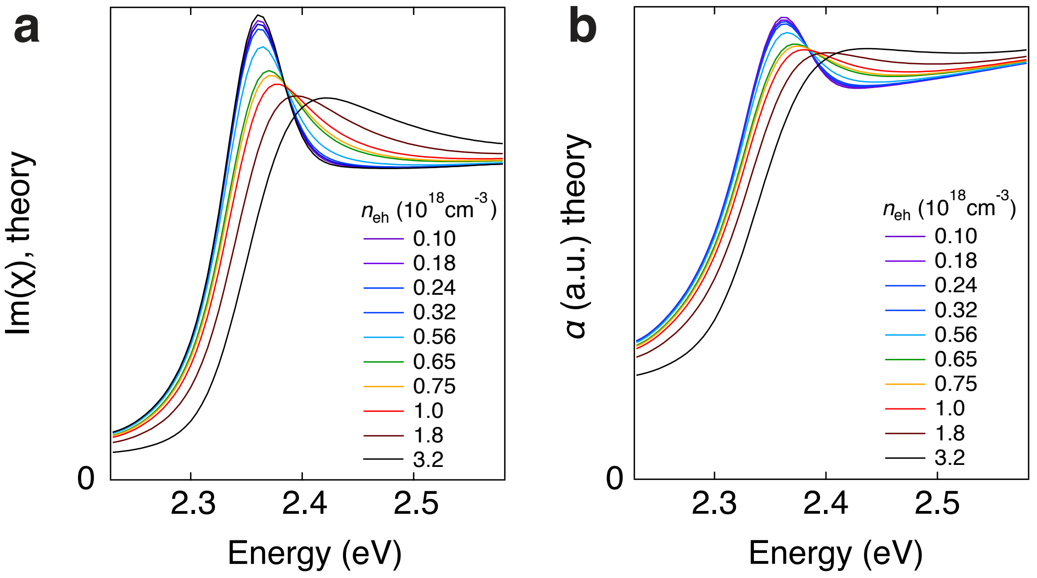

Supplementary Figure 11: Imaginary part of the optical susceptibility (Im(χ), (**a**)) and absorption spectrum (α, (**b**)) of $CH_3NH_3PbBr_3$ in the presence of increasing carrier densities (0.1 to 3.2×10^{18} cm^{-3}) as calculated with the SBE.

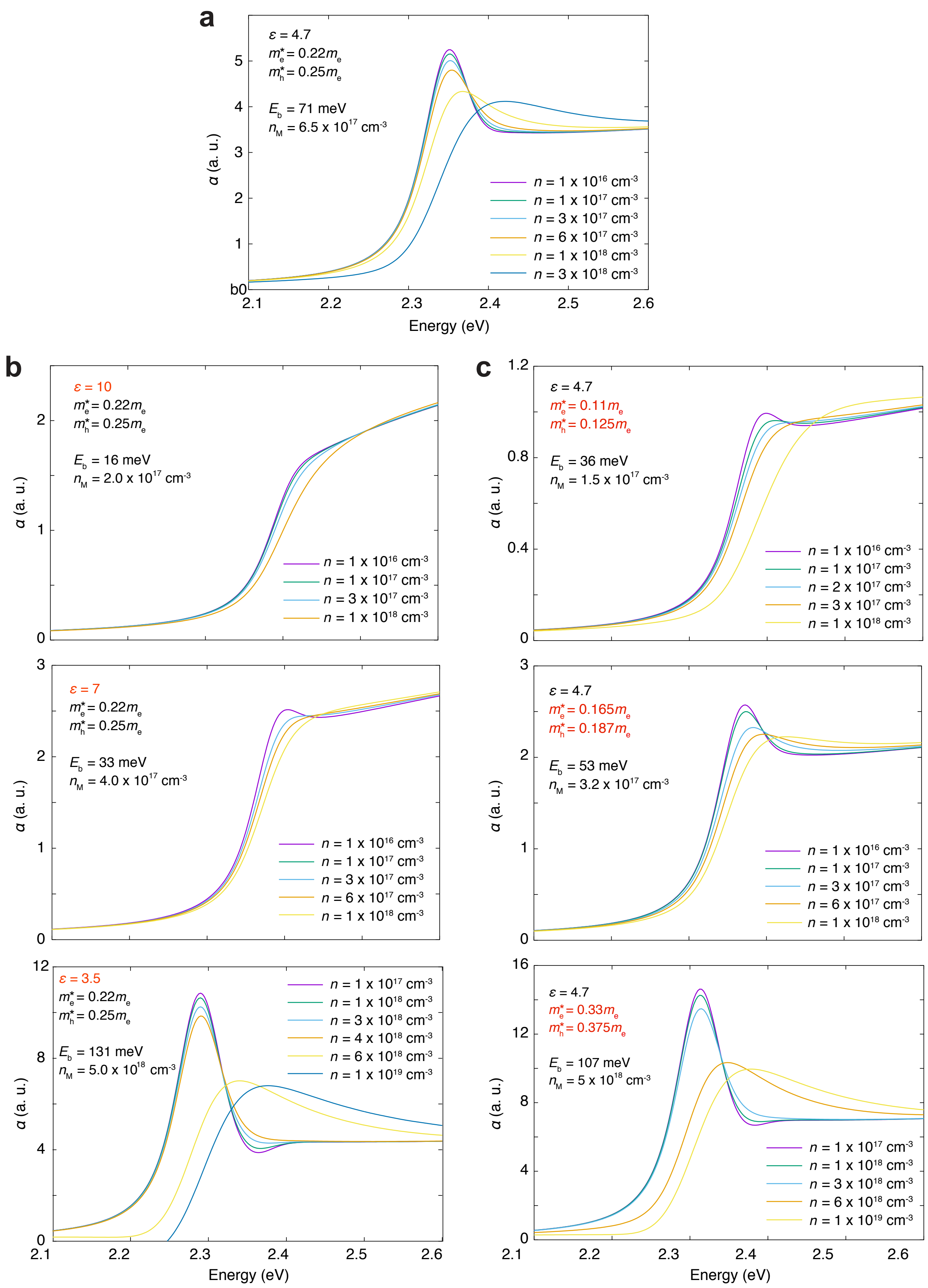

Supplementary Figure 12: Theoretical absorption spectra of $CH_3NH_3PbBr_3$ in the presence of increasing carrier densities (n), and for different combination of dielectric constant (ε) and carrier effective masses (m_e^*, m_h^*) as calculated with the SBE. (**a**) Theoretical spectra corresponding to the reference parameters. (**b-c**) Theoretical spectra for decreasing value of ε (b), and increasing m_e^*, m_h^* (c), both resulting in increasing E_b and n_M.

Supplementary Table 1: Amplitude (A), linewidth (Γ) and peak energy (E_0) for the Lorentz oscillator and the five TL oscillators. Other parameters are $\varepsilon_\infty = 2.02561 \pm 0.0350$ and $E_\mathrm{g} = 2.2783 \pm 0.0044 eV$.

Oscillator	A	Γ	$E_\mathbf{0}$
Lorentz	0.2483 ± 0.0190	0.0914± 0.0028	2.3514 ± 0.0008
TL1	211.9 ± 0.0889	0.1728 ± 0.0608	2.2464 ± 0.0203
TL2	17.83 ± 0.0011	1.1128 ± 0.0416	3.2122 ± 0.0068
TL3	5.20 ± 0.0004	0.6270 ± 0.0246	3.9441 ± 0.0045
TL4	6.81 ± 0.0003	0.8305 ± 0.0147	4.3677 ± 0.0070
TL5	6.44 ± 0.0005	1.4348 ± 0.1049	6.2195± 0.0532

Supplementary Note 1 : Elliott theory of excitonic absorption

In order to estimate the binding energy (E_b) of Wannier excitons hosted by a semiconductor, a powerful approach is to fit the band-edge absorption spectrum with the Elliott formula [1].

According to Elliott theory, the absorption coefficient can be expressed as [2]

$$\alpha(\omega) = \alpha_0^{3D}\left(\sum_{j=1}^{\infty} a_j + a_\mathrm{c}\right) = \alpha_0^{3D}\frac{\hbar\omega}{E_\mathrm{b}}\left[\sum_{j=1}^{\infty}\frac{4\pi}{j^3}\delta\left(\Delta+\frac{1}{j^2}\right)+\Theta(\Delta)\frac{\pi e^{\frac{\pi}{\sqrt{\Delta}}}}{\sinh\left(\frac{\pi}{\sqrt{\Delta}}\right)}\right], \tag{1}$$

where Δ and E_b are defined as

$$\Delta = \frac{\hbar\omega - E_\mathrm{g}}{E_\mathrm{b}}, \qquad E_\mathrm{b} = \frac{\mu e^4}{2\varepsilon^2\hbar}. \tag{2}$$

Here, j is a positive integer; a_j and a_c are the contribution from the j-exciton state and the continuum, respectively; μ is the exciton reduced mass; $\delta(x)$ is the delta function; $\Theta(x)$ is the step function; ε the dielectric constant; E_g the value of the single-particle bandgap; α_0^{3D} is the three-dimensional joint density of state prefactor, which reads

$$\alpha_0^{3D} = \frac{2\pi|d_\mathrm{cv}|^2}{\hbar\varepsilon_0 n_\mathrm{b} c}\frac{4\pi}{(2\pi a_\mathrm{B})^3}, \tag{3}$$

where d_cv is the transition dipole moment, ε_0 is the vacuum permittivity, n_b is the background refractive index, c is the speed of light, a_B the exciton Bohr radius. We convolute Supplementary Equation (1) with a hyperbolic secant function to account for a phenomenological broadening $\mathit{\Gamma}$, and introduce a term to correct for the non-parabolic band dispersion (in SI units).

The final expression for $\alpha(\omega)$ reads

$$\alpha(\omega) = \left[\frac{|d_\mathrm{cv}|^2(2\mu)^{3/2}}{\varepsilon_0\hbar n_\mathrm{b} c}\right]E_\mathrm{b}^{1/2}\hbar\omega\left[\sum_{j=1}^{\infty}\frac{2E_\mathrm{b}}{j^3}\mathrm{sech}\left(\frac{\hbar\omega - E_\mathrm{g} + E_\mathrm{b}/j^2}{\mathit{\Gamma}}\right)+\right.$$

$$\left.+\int_{E}^{E_\mathrm{g}}\mathrm{sech}\left(\frac{E-x}{\mathit{\Gamma}}\right)\frac{1}{1-e^{-2\pi\sqrt{\frac{E_\mathrm{b}}{x-E_\mathrm{g}}}}}\frac{1}{1-\frac{8\mu b}{\hbar^3}(x-E_\mathrm{g})}dx\right]. \tag{4}$$

The above function is used to fit the experimental absorption spectrum (blue dots, Fig. 1c in the main

text), yielding $E_\mathrm{b} = 71$ meV, $\mathit{\Gamma} = 34$ meV, $E_\mathrm{g} = 2.42$ eV. The prefactor in brackets in Eq. 4 is treated as a multiplying constant.

Supplementary Note 2 : Estimate of the critical Mott density

In the presence of high carrier densities, Coulomb screening (CS) is expected to reduce E_b until complete disappearance of the e-h interaction. In the literature, there exist several criteria to estimate the critical density n_M at which the transition occurs. In this Note, we apply such criteria to $CH_3NH_3PbBr_3$ single crystals, and compare the estimated n_M with the values previously reported for $CH_3NH_3PbI_3$ at room temperature (10^{16}-10^{19}cm^{-3}) [3, 4, 5]. In general, these criteria are based on the comparison between the exciton Bohr radius a_B and the screening length of the free carriers. As screening mechanisms are involved, knowledge of the material's dielectric constant is necessary. However, because in hybrid lead-halide perovskites the dielectric function changes by order of magnitudes with photon energy, the choice of the correct $\varepsilon(\omega)$ is critical.

Following [5], we determine the value of the effective dielectric constant ε_{eff} from the expression

$$E_b = \frac{\mu e^4}{2\hbar(\varepsilon_{\text{eff}}\varepsilon_0)^2}. \tag{5}$$

From the experimental value of E_b = 71 meV (see Supplementary Note 1 and Figure 1c), and taking $m_e^* = 0.22m_e$, $m_h^* = 0.25m_e$, $\mu = 0.117m_e$ as determined from previous GW calculations [6], we estimate $\varepsilon_{\text{eff}} = 4.73$ and $a_B = 2.14$ nm. By using different Mott criteria as expressed in Supplementary Equations (6)–(7), we find n_M for a $CH_3NH_3PbBr_3$ single crystal

$$n_M = 1.19^2 \frac{k_B T}{2a_B^3 E_b} = 2.01 \cdot 10^{18}\text{cm}^{-3} \tag{6}$$

$$n_M = a_B^{-3} \frac{\pi 1.19^6}{4^3 \cdot 3} \left(\frac{m_e m_h}{(m_e + m_h)^2} \right)^3 = 7.31 \cdot 10^{16}\text{cm}^{-3} \tag{7}$$

Similarly to what reported for $CH_3NH_3PbI_3$, the variability of n_M spans two orders of magnitude, making the estimation highly dependent on the criteria used and the related assumptions. In the following Note, we will provide the details of an alternative method that accounts for many-body effects and allows for a precise estimate of n_M.

Supplementary Note 3 : Theory of ionization equilibrium

In this Supplementary Note, we present the details of the theory of ionization equilibrium, a many-body theory that provides a precise estimate of n_M in the exciton-plasma conglomerate. Such an approach overcomes the limitation of the Mott criteria used in Supplementary Note 2, which suffer from high variability and usually yield an over- or underestimation of n_M by one/two orders of magnitude. Following [7] and [8], we start from the general expression for the carrier density n_a of the a species (electron or hole)

$$n_a(\mu_a, T) = \frac{i\hbar}{\mathcal{V}} \int_{-\infty}^{\infty} \frac{d\omega}{2\pi} \sum_{\mathbf{k}\sigma} f^a(\omega) A^a_{\mathbf{k}\sigma}(\omega) \ , \tag{8}$$

where $f^a(\omega)$ denotes the Fermi distribution function depending on the chemical potential μ_a and the temperature T, and $\mathcal{V}$ is the crystal volume. The spectral function $A^a_{\mathbf{k}\sigma}(\omega)$ of the single-particle state $|\mathbf{k}\sigma a\rangle$ with spin σ is given by

$$A^a_{\mathbf{k}\sigma}(\omega) = 2i\mathrm{Im}\, \frac{1}{\hbar\omega - \varepsilon^{0,a}_{\mathbf{k}\sigma} - \Sigma^{\mathrm{ret},a}_{\mathbf{k}\sigma}(\omega)} \ . \tag{9}$$

The retarded self-energy $\Sigma^{\mathrm{ret},a}_{\mathbf{k}\sigma}(\omega)$ accounts for many-particle effects giving rise to renormalizations of the single-particle band structure $\varepsilon^{0,a}_{\mathbf{k}\sigma}$ as well as contributions of bound states. For a given self-energy, the inversion of Supplementary Equation (8) yields $\mu_a(n_a, T)$ for each species and therefore any thermodynamic property of the system in the grand canonical formulation. In the limit of small quasiparticle damping ($\mathrm{Im}\,\Sigma^{\mathrm{ret},a} \ll \mathrm{Re}\,\Sigma^{\mathrm{ret},a}$), the spectral function can be expanded in linear order of $\mathrm{Im}\,\Sigma^{\mathrm{ret},a}$ yielding the carrier density in so-called extended quasiparticle approximation

$$\begin{aligned} n_a(\mu_a, T) &= \frac{1}{\mathcal{V}} \sum_{\mathbf{k}\sigma} f^a(\varepsilon^a_{\mathbf{k}\sigma}) - \frac{1}{\mathcal{V}} \sum_{\mathbf{k}\sigma} \int_{-\infty}^{\infty} \frac{d\omega}{2\pi} \frac{2}{\hbar} \mathrm{Im}\,\Sigma^{\mathrm{ret},a}_{\mathbf{k}\sigma}(\omega)\, [f^a(\varepsilon^a_{\mathbf{k}\sigma}) - f^a(\omega)] \frac{d}{d\omega} \frac{\mathcal{P}}{\omega - \varepsilon^a_{\mathbf{k}\sigma}/\hbar} \\ &= n_a^{\mathrm{QP}} + n_a^{\mathrm{corr}} \ , \end{aligned} \tag{10}$$

where the quasiparticle energy $\varepsilon^a_{\mathbf{k}\sigma}$ is given by $\varepsilon^a_{\mathbf{k}\sigma} = \varepsilon^{0,a}_{\mathbf{k}\sigma} + \mathrm{Re}\,\Sigma^{\mathrm{ret},a}_{\mathbf{k}\sigma}(\varepsilon^a_{\mathbf{k}\sigma})$ and $\mathcal{P}$ denotes the Cauchy principal value. The total density is divided into contributions from quasi-free particles and correlated particles, the latter being either in bound or scattering many-particle states. To evaluate the

expressions (9) and (10), a suitable approximation for the self-energy $\Sigma^{\mathrm{ret},a}(\omega)$ is needed. The real and imaginary parts of Σ determine the quasiparticle energies and the correlated part of the carrier density, respectively. An appropriate choice is the screened ladder approximation [9, 10, 11] $\Sigma(\omega) = \Sigma^{\mathrm{H}} + \Sigma^{\mathrm{GW}}(\omega) + \Sigma^{\mathrm{T}}(\omega)$, which takes into account screening of Coulomb interaction due to excited carriers as well as the formation of bound two-particle states and consists of Hartree, GW and T-matrix contributions. We assume that renormalizations due to the Hartree self-energy are small compared to exchange and correlation effects. In the T-matrix contribution, we neglect exchange terms and assume static screening so that the T-matrix depends only on one instead of three frequency arguments. Expressing the T-matrix by its so-called bilinear expansion into two-particle eigenstates leads to the final expression for the carrier density [7, 10]

$$n_a(\mu_a, T) = \frac{1}{\mathcal{V}} \sum_{\mathbf{k}\sigma} f^a(\varepsilon^a_{\mathbf{k}\sigma}) + \frac{1}{\mathcal{V}} \sum_{b \neq a} \sum_{\sigma\sigma'} \sum_{\nu\mathbf{Q}} n^{\mathrm{B}}_{ab}(E^{\sigma\sigma'}_{\nu\mathbf{Q}}) = n^{\mathrm{GW},a}_{\mathrm{free}} + n_{\mathrm{X}}. \tag{11}$$

The total carrier density separates into contributions from quasi-free carriers and from carriers bound as excitons. We identify excitons as those two-particle states $|\nu\sigma\sigma'\mathbf{Q}\rangle$ which have eigenenergies $E^{\sigma\sigma'}_{\nu\mathbf{Q}}$ below the renormalized quasiparticle gap, while contributions from scattering states above the gap are neglected for simplicity. ν describes the relative motion of electron and hole, whereas $\mathbf{Q}$ denotes the total momentum of the exciton. While unbound carriers enter the carrier density via Fermi distribution functions, exciton states are populated according to the Bose distribution function $n^{\mathrm{B}}_{ab}(\omega) = \left[\exp(\beta(\hbar\omega - \mu_a - \mu_b)) - 1\right]^{-1}$ depending on the chemical potentials of both carrier species.

For a specific material, the ionization equilibrium has to be computed numerically. The electron and hole chemical potentials are determined by adapting the Fermi functions $f^a(\varepsilon^a_{\mathbf{k}\sigma})$ of electrons and holes to a given density of quasi-free carriers at the quasiparticle energies $\varepsilon^a_{\mathbf{k}\sigma}$. As the chemical potentials also enter the bound-carrier density via the Bose function n^{B}_{ab}, Supplementary Equation (11) represents an implicit equation for the fraction of quasi-free carriers $\alpha_a = n^a_{\mathrm{free}}/n_a$, which has to be solved self-consistently with the quasiparticle energies in GW approximation, see Supplementary Equation (22), and the bound-state energies $E^{\sigma\sigma'}_{\nu\mathbf{Q}}$.

The quasiparticle renormalizations are evaluated using only the quasi-free carrier density. In par-

ticular, the dynamical screening is treated in the random-phase approximation with a macroscopic Lindhard dielectric function [12], taking into account contributions from unbound carriers only. A similar contribution to the dielectric function emerges for bound carriers involving bosonic exciton population functions and energies [13]. However, due to the composite character of excitons, this screening contribution is not metal-like but relies on the weak exciton dipole moment. Due to the almost perfect neutrality of excitons, we assume that in all cases the exciton contribution can be safely neglected compared to the plasma contribution.

To further simplify the procedure, we exploit the fact that shifts of excitonic resonances are naturally much smaller than bandgap shifts, which is due to compensation effects between gap shrinkage and E_b reduction. Hence, we assume that the absolute spectral position of the exciton remains the same even in the presence of carriers. The net effect is the reduction of E_b until it is fully consumed at n_M, above which only unbound carriers are present. The exciton ionization ratio (α_eh, defined in the main text) as a function of the excitation density as calculated from the theory of ionization equilibrium is shown in Figure 1d (red dots). Here, the vertical line indicates the Mott critical density, found at $n_\mathrm{M} \approx 5 \times 10^{17}$ cm^{-3}.

Saha-Langmuir equation

The mass-action law (Saha-Langmuir equation [14]) treats free carriers and excitons as two distinct species in thermodynamic equilibrium, where a balance exists between dissociation and recombination events. In the Saha-Langmuir equation, the ionization ratio $\alpha_\mathrm{eh,SL}$ is influenced by E_b and by the excitation density n. $\alpha_\mathrm{eh,SL}$ is defined by [15]

$$\frac{\alpha^2_\mathrm{eh,SL}}{1-\alpha_\mathrm{eh,SL}} = \frac{1}{n\lambda_T^3} \exp\left(\frac{-E_\mathrm{b}}{k_\mathrm{B}T}\right), \tag{12}$$

where $\lambda_T = h/\sqrt{2\pi\mu k_\mathrm{B}T}$ is the thermal de Broglie wavelength. The resulting curve is shown in Figure 1d (solid line) in the main text.

Supplementary Note 4 : Determination of the photogenerated carrier density

In order to explore the photophysics of $CH_3NH_3PbBr_3$ crystals in the high-density regime, it is crucial to accurately estimate the experimental electron-hole (e-h) density, n, created by the pump pulse. This quantity can be expressed as

$$n = (1 - R)\frac{F}{h\nu\lambda_\mathrm{p}}, \tag{13}$$

where F is the pump fluence, $h\nu$ is the pump photon energy, $\lambda_\mathrm{p} = 1/\alpha$ the light penetration depth in the material, and R is the reflectivity of the sample. All parameters are evaluated at the pump photon energy (3.10 eV).

The uncertainty in n can be estimated by propagating the uncertainty in the variables entering Supplementary Equation (13), namely the absorption/reflection coefficients of the sample and the laser parameters. Moreover, as the choice of the excitation volume geometry is arbitrary, we discuss the approximations introduced for the calculation of the excitation spot size. Finally, to ensure the validity of Supplementary Equation (13), the presence of saturation and fast recombination effects have to be excluded. We address these issues in detail in the following.

Absorption and reflection coefficients:

The estimates of the absorption coefficient, as well as the amount of reflection from the sample surface, are based on measured data of spectroscopic ellipsometry, which is the most accurate experimental technique currently available to determine the real and imaginary parts of the dielectric function for any insulator above its fundamental gap. Our spectroscopic ellipsometry data (shown in Supplementary Figure 2) were benchmarked on different single crystals of $CH_3NH_3PbBr_3$ and the absorption/reflectance spectra obtained directly from the measured optical quantities without the need of a Kramers-Kronig analysis. Since the error on the measured ellipsometry angles Ψ and Δ is less than 0.1%, the uncertainty in R, α, and λ_p remains well below 1%.

Incident laser fluence:

The average incident laser fluence F (measured in μJ cm^{-2}) is defined as $F = P/(r \cdot A)$, where P is the laser power, r the repetition rate of the laser system, and A the laser spot size. The impinging laser power P is measured accurately by using an ultraviolet-extended ultra-sensitive Si photodiode, and is corrected by taking into account the loss due to the reflection of the cryostat window. For our photodiode, the uncertainty of the power measurement at a photon energy of 3.10 eV is equal to $\delta P = \pm 4\%$. The measurement of the spot size is performed using a camera-based beam profiling system consisting of a camera and analysis software. Here, the uncertainty in the width measurement is $\delta w = \pm 2\%$. Error propagation from w and P yields to an uncertainty in the incident laser fluence of $\delta F = \sqrt{2\delta w + \delta P} \approx 5\%$.

Choice of the excitation volume:

Although the uncertainty in n related to the laser parameters and absorption/reflection of the sample remains below 6%, an additional source of variation in the estimated e-h density originates from the arbitrary choice of the excitation volume, which appears as the product $A\lambda_{\text{p}}$ in Supplementary Equation (13). In agreement with the approach used in literature, as excitation volume we consider a cylinder whose height is equal to the light penetration depth λ_{p} (*i.e.*, the depth at which the intensity of the radiation is decreased by approximately $1/e = 37\%$ of its initial value). This choice of λ_{p} is justified by the similarity between the absorption coefficient of the pump and probe in the exciton region ($5 \div 10 \mu\text{m}^{-1}$); thus, we can reasonably consider the same penetration depth for the pump and probe energies. On the other side, the choice of the spot size A deserves more attention. In Supplementary Figure 3a, we simulate the gaussian excitation beam used in our experiment. Conventionally, the spot size diameter is approximated by the full width at half maximum (FWHM) of the gaussian profile

$$A := \pi \left(\frac{\text{FWHM}}{2} \right)^2 , \tag{14}$$

Supplementary Figure 3b shows the results of such an approximation by comparing the profiles of the two-dimensional intensity distributions of the gaussian beam (red curve) and the FWHM cylinder. In the former case, since the total intensity (*i.e.* the total volume under the gaussian surface) is contained

in a smaller base, the avarage peak intensity is almost 1.5 times the peak intensity of the gaussian. However, the validity of this approximation depends on the relative size of the probe beam with respect to the pump, since a small probe will be able to probe *locally* the photoexcited surface and provide a more precise estimation of the e-h density. This concept is illustrated in Supplementary Figure 3c,d, where we compare the simulated profiles of the pump and probe beams used in the experiment. When the two laser beams are perfectly overlapped, being the probe much narrower than the pump, the probed area will coincide with the most intense part of the gaussian, whose intensity is almost 1.5 times smaller than the one resulting from the FWHM approximation. With the latter being the approximation we use in our study, the declared excitation densities may *exceed* the actual densities by a factor 1.5-2.

Therefore, by considering all sources of uncertainty presented above, we conclude that the densities reported in the manuscript will differ from the actual densities by a factor no greater than 1.5-2. Such an uncertainty does not significantly affect the comparison of the experimental and theoretical spectra, nor influence the discussion and conclusion of our study.

Saturation behaviour and fast decay:

We can ensure the direct proportionality between laser beam intensity and e-h excitation density if saturation effects can be ruled out. The absence of a saturation behaviour up to 40 μJ cm^{-2} (1×10^{18}cm^{-3}–10^{19}cm^{-3}) can be appreciated by direct inspection of the raw data in Supplementary Figure 4, summarized for clarity in Supplementary Figure 5. In panel a, we compare the $\Delta R/R$ signal for the three excitation fluences at 500 fs (*i.e.* the time at which the response is maximum). The $\Delta R/R$ value corresponding to the positive and negative peaks are plotted respectively in Supplementary Figure 5b,c, and show that the size of $\Delta R/R$ still increases with increasing incident fluence. Despite the emergence of many-body effects and related nonlinearities in the signal at higher fluences, the above results rule out the involvement of any significant saturation behaviour, and ensures a direct proportionality between the laser intensity profile and the photogenerated e-h density distribution. Saturation effects start appearing close to the largest carrier density explored, similar to the trend reported previously [16].

Finally, it is straighforward to rule out the presence of a substantial and fast decay of the charge carriers. Transient reflectivity and absorption measurements with high time resolution ($\ll$ 100 fs) are capable of mapping the dynamics of the two-particle spectral function right after photoexcitation, unlike photoluminescence measurements that are mainly sensitive to e-h annihilation [17]. In the specific case of hybrid perovskites, the rise of the transient reflectivity/absorption signal ($\approx$ 500 fs) denotes the time it takes the photoexcited carriers to complete intraband cooling (which occurs prior electron-hole recombination). This timescale is relatively long due to the presence of a phonon bottleneck that establishes right after photoexcitation [18, 19].

Supplementary Note 5 : Reflectivity lineshape analysis

In this Supplementary Note, we present the details of the lineshape analysis performed on the static complex dielectric function and the transient reflectivity spectra measured on $CH_3NH_3PbBr_3$ single crystals. The goal of this analysis is retrieving the time-dependent complex dielectric function ($\varepsilon(\omega, t)$ = $\varepsilon_1(\omega, t)$ + i$\varepsilon_2(\omega, t)$) and using it to calculate the optical quantity of interest in the most accurate way.

Comparison between Kramers-Kronig transformations and reflectivity lineshape analysis

An approach that is commonly followed to extract the real and imaginary part of the dielectric function relies on the use of the normal-incidence reflectivity $R(\omega)$ spectrum and the Kramers-Kronig transformations (KKT). Here we describe why this approach is inaccurate for our purposes, justifying the need of a more precise linesape analysis [20, 21]. Specifically, the KKT require the knowledge of the frequency-dependent phase shift angle of the sample $\theta(\omega)$, which is expressed by an integral of the function $R(\omega)$ over the $\omega = [0, \infty)$ frequency range. Since $R(\omega)$ has to be extrapolated outside the experimentally-accessible frequency range, approximations are required at both extremes. Therefore, this method is only suitable when $R(\omega)$ is negligible outside the measured range, which is not the case for $CH_3NH_3PbBr_3$ (see Supplementary Figure 2). For our transient reflectivity spectra, the uncertaintly introduced by the approximation would be comparable or larger than the laser-induced changes, preventing the iterative KKT from tracking precisely the evolution of the exciton peak and the above-gap absorption [20].

In contrast, the lineshape analysis implemented in the following is based on the equilibrium $R(\omega)$ spectrum (determined in a direct way via ellipsometry) and a Kramers-Kronig-constrained fitting of its time-varying version $R(\omega, t)$ (which is recalculated at each iteration). The photoinduced changes in $R(\omega, t)$ are modelled through *simultaneous* changes in $n(\omega)$ and $\kappa(\omega)$, whose steady-state functions are also known *a priori* via the ellipsometry experiment, and which are dynamically coupled by the KKT during the fit [20]. Therefore, unlike the KKT approach, our method only requires the punctual values of $n(\omega)$ and $\kappa(\omega)$ at a given frequency (see Equations (1) and (2) in the Methods section). As no integrals are calculated in this procedure, no approximations are used, providing a robust way to retrieve any optical quantity with the highest level of accuracy.

Lineshape analysis

In our approach, we simultaneously fit the experimentally-determined $\varepsilon_1(\omega)$ and $\varepsilon_2(\omega)$ with a model comprising one Lorentz and five Tauc-Lorentz (TL) oscillators describing the excitonic resonance and the continuum, respectively. The fit of $\varepsilon_2(\omega)$ is limited to the region 2.22 eV - 5.50 eV due to artifacts of the measurements at lower energies. The analytical expression of $\varepsilon_1(\omega)$ and $\varepsilon_2(\omega)$ of TL functions can be found in Ref. [22]. The results of the fit are shown in Supplementary Figure 2a as solid lines. The oscillator parameters are listed in Supplementary Table 1.

The transient reflectivity signal is defined as

$$\frac{\Delta R}{R}(\omega, t) = \frac{R(\omega, t) - R_{\mathrm{s}}(\omega)}{R_{\mathrm{s}}(\omega)}, \tag{15}$$

where $R(\omega, t)$ is the reflectivity at the pump-probe delay t and $R_{\mathrm{s}}(\omega)$ is the equilibrium reflectivity spectrum. We assume the latter to be equal to the reflectivity extracted from the ellipsometry data as explained in the Methods section, and shown in Supplementary Figure 2b.

Finally, we combine our steady-state and time-resolved data and obtain the time evolution of the reflectivity $R(\omega, t)$

$$R(\omega, t) = R_{\mathrm{s}}(\omega) \times \frac{\Delta R}{R}(\omega, t) + R_{\mathrm{s}}(\omega) \tag{16}$$

The resulting spectra are shown in Supplementary Figure 6. Via the TL model of the reflectivity, we can directly relate the changes in reflectivity to the variation of the oscillator parameters. We consider the absolute reflectivity spectra of Supplementary Figure 6 and fit them iteratively within the TL model. As starting parameters, we use the steady-state ones; leaving the Lorentz and the first TL oscillator free to vary is sufficient to reproduce well the spectra at all times delays. This approach allows us to track the temporal evolution of the Lorentz oscillator parameters, which are presented in Supplementary Figure 7.

Once the evolution of the model parameters is characterized, the dynamics of any optical quantity of interest becomes accessible. In particular, we can extract the time-dependent absorption coefficient $\alpha(\omega, t)$. Its evolution during the rise and decay of the response is shown in Supplementary Figure 8.

In the first 500 fs, we observe a sizeable decrease of the exciton oscillator strength, accompanied by a slight redshift of its peak energy. Remarkably, besides this renormalization, the exciton lineshape remains well resolvable even at this high excitation density.

The well-resolved excitonic resonance in the $CH_3NH_3PbBr_3$ optical spectrum allows to qualitatively describe the first Lorentz oscillator in terms of a Wannier exciton, and rationalize the single-particle and many-body effects involved in the observed exciton renormalization of Supplementary Figure 7. The decrease in the oscillator strength (Supplementary Figure 7a) can be associated with band filling of the single-particle states contributing to the exciton. In $CH_3NH_3PbBr_3$, the band filling contribution to the exciton bleaching is caused by the relaxation of e-h pairs to the band edges at the R point of the Brillouin zone [23, 6]. The timescale for the decrease ($\approx$500 fs) is related to the intraband cooling dynamics, which is governed by the emission of longitudinal-optical phonons via the Fröhlich interaction [24, 18, 25]. The shift in the exciton energy ΔE_{x} results instead from the simultaneous action of single- and many-particle renormalization effects. Specifically, from Supplementary Figure 7b we detect a redshift from 6 meV to 10 meV. This indicates that bandgap renormalization (BGR) is present, which reduces the single-particle energy through the modification of the exchange-correlation potential [26]. The increase in exciton linewidth (Supplementary Figure 7c) suggests that long-range CS is also at play, as the total (delocalized and trapped) photoexcited carrier density is expected to screen the e-h Coulomb attraction and reduce the exciton lifetime [27].

Supplementary Note 6 : Absorption lineshape analysis

In the lineshape analysis presented in Supplementary Note 5, the choice of the model for the dielectric function is not unique, and for each model the characteristics of the oscillators will be different. For this reason, any attempt to extract E_{b} from the TL oscillators would be rather inaccurate. Therefore, more refined models like Elliott theory (see Supplementary Note 1) are needed to estimate the exciton parameters. For this reason, in this Supplementary Note we perform additional analysis by applying Elliott theory iteratively on the time-resolved absorption spectra.

To account for the change in the exciton lineshape in the nonequilibrium case, we modify Supplementary Equation (4) by multiplying the exciton part by a factor OS, which accounts for changes in exciton oscillator strength (primarily due to exciton bleaching). OS, E_{b}, $\mathit{\Gamma}$, and E_{g} are free parameters in the fit. The iterative fit of $\alpha(\omega, t)$ via the modified Elliott model results in the time-evolution of the significant parameters, shown in Supplementary Figure 9. In addition, we compute the exciton peak position ($\Delta E_{\mathrm{X,Elliott}} = \Delta E_{\mathrm{g}} - \Delta E_{\mathrm{b}}$) and compare it with $\Delta E_{\mathrm{X,TL}}$ resulting from the TL fit (Supplementary Figure 10).

In the latter figure, we notice an excellent agreement between the exciton peak position tracked by the two models, which both show a redshift of 5-10 meV in the first 500 fs, followed by a blueshift and stabilization around a constant energy value which depends on the excitation density. This bi-phasic behaviour is further confirmed by the visual inspection of the absorption spectra in Supplementary Figure 8 in the rise and the decay of the response. We focus now on the evolution of OS, E_{b}, $\mathit{\Gamma}$, and E_{g}. The exciton OS decreases within 500 fs, and recovers with a bi-exponential trend (Supplementary Figure 9a). A similar temporal evolution is followed by the renormalization of the single-particle gap energy E_{g} (Supplementary Figure 9c). In contrast, a different temporal trend characterizes the exciton E_{b} and the exciton linewidth $\mathit{\Gamma}$, which respectively undergo a slight drop and increase before stabilizing around a constant value (Supplementary Figs 9b,d). At high excitation densities, similar but more pronounced trends are observed, with differences with respect to the trends observed in the Tauc-Lorentz reflectivity fit. In particular, ΔOS becomes positive at higher fluences, which is in contrast with the absolute decrease in the peak observed in Supplementary Figure 8. We associate this feature to the persistence of the exciton peak, which instead is expected to reduce following the decrease

in E_b (see Supplementary Equation (4)). Nevertheless, we stress that the trends in Supplementary Figure 9 have just a qualitative meaning: in fact, the Elliott formula of Supplementary Equation (4) has a simple form only in the limit of *low* excited carrier density. At high excitation densities towards the Mott transition, the two-particle wave functions will be strongly modified by many-body effects, deviating from hydrogen-like wave functions. Therefore, the trend for the oscillator strength could be affected by artefacts due to the inappropriate form of the fit function that is used. Moreover, the low-density Elliott formula does not describe the excitonic enhancement. Despite these limitations, the above analysis is to our knowledge unique in combining the iterative Elliott fit to time-resolved spectra, and represents a novel approach to investigate the qualitative behaviour of the exciton parameters in semiconductors.

Our observations point towards the persistence of the Wannier exciton peak and the formation of Mahan excitons above n_M in our hybrid perovskite single crystals. While the term Mahan exciton was initially discussed in the context of degenerate (i.e. chemically-doped) semiconductors and metals [28, 29], it has subsequently been extended to photo-doped semiconductors [30, 31, 32, 33]. This is because the latter share the same description of chemically-doped semiconductors in terms of Bethe-Salpeter and semiconductor Bloch equations (SBE) [34, 35]. The main difference lies in the fact that one scenario involves electron and hole occupancies as nonlinearity in the two-particle equation, whereas the other scenario relies on only one of them. In order for the same description to hold, the electrons and holes in the photo-doped semiconductor must have reached quasi-equilibrium, which implies that the Mahan exciton feature has to be well defined even after the intraband cooling is complete (as in our data). Band filling can be stronger in the photoexcitation case, possibly leading to population inversion, and separate electron and hole chemical potentials have to be defined instead of one global chemical potential. However, there is a window of excitation densities between n_M and population inversion where the situation is analogous to the simple chemical-doping case. Many-body effects such as BGR and CS due to intraband polarization still play a role.

We remark that none of these conventional single-particle (band filling) and many-body effects (BGR, CS) can account for the enhancement of the absorption continuum above the Wannier exciton peak. Similarly, we can rule out a scenario in which e-h Cooper pairs are responsible for the Fermi-edge

singularity [36]. In the presence of condensed e-h Cooper pairs, a nonequilibrium (Keldysh) excitonic insulator is expected to form [37]. When such an exciton condensation occurs in nonequilibrium conditions, the bands of the semiconductor undergo an electronic structure reconstruction and many-body gaps open at the quasi-Fermi surfaces of electrons and holes (analogous to the many-body gap that opens at the Fermi surface of a metal in the case of a superconducting instability) [38]. The size of these many-body gaps depends on the value of E_b and their presence quenches any Fermi-edge singularity in the two-particle spectral function measured by optical spectroscopy. The same scenario is expected when the e-h Cooper pairs are preformed above the transition temperature for exciton condensation (and a Bose-Einstein condensation mechanism is thus at play instead of a Bardeen-Cooper-Schrieffer scenario). The gaps would still have a finite size, but the macroscopic phase coherence would be lost. This concept is widely explored in the physics of superconductors [39], where preformed pairing leads to a finite superconducting gap amplitude in the single-particle spectral function but long-range phase coherence is lost. Similar results were obtained for candidate equilibrium excitonic insulators that may lie close to the Bose-Einstein condensation regime [40]

Supplementary Note 7 : Semiconductor Bloch equations

In the following, we present in detail the solution of the SBE, which yield the theoretical absorption spectra of $CH_3NH_3PbBr_3$ in the presence of photoexcited carriers. The absorption of a semiconductor is obtained from its linear response to a classical electric field $\mathbf{E}(t)$ [2]. The electric field drives a macroscopic polarization of the medium that is composed of the microscopic inter-band polarizations $\psi^{\text{he}}_{\mathbf{k}}(t) = \langle a^{\text{h}}_{\mathbf{k}} a^{\text{e}}_{\mathbf{k}} \rangle(t)$, with the annihilation operator $a^{\text{e/h}}_{\mathbf{k}}$ of electrons and holes, according to $\mathbf{P}(t) = \frac{1}{\mathcal{V}} \sum_{\mathbf{k},\text{eh}} \psi^{\text{he}}_{\mathbf{k}}(t)(\mathbf{d}^{\text{eh}}_{\mathbf{k}})^* + \text{c.c.}$. Here, $\mathcal{V}$ is the crystal volume and $\mathbf{d}^{\text{eh}}_{\mathbf{k}}$ are dipole matrix elements describing the efficiency of light-matter coupling depending on the momentum $\mathbf{k}$. In the regime of weak excitation, the macroscopic polarization is linear in the electric field. This allows to extract the response of the medium as a pure material property in the form of a susceptibility that is independent of the electric field

$$\chi(\omega) = \frac{P(\omega)}{\varepsilon_0 E(\omega)}\,, \tag{17}$$

where we consider an isotropic susceptibility χ. To obtain the frequency-dependent response of the medium, we use the Fourier transform of the macroscopic polarization $\mathbf{P}(t)$, assuming the medium to be in a quasi-equilibrium state. In this picture, any excited carriers that are generated by pumping the active medium relax to quasi-equilibrium distributions at a certain temperature and carrier density. The absorption at these parameters is then probed by a weak electric test field.

Applying the technique of nonequilibrium Green functions, the equation of motion for microscopic inter-band polarizations known as SBE can be derived [41, 42]. As shown in [43], the SBE are transformed into frequency space taking into account many-body effects due to excited carriers on a GW-level

$$\begin{aligned} &\Big(\hbar\omega - \varepsilon^{\text{HF,h}}_{\mathbf{k}} - \varepsilon^{\text{HF,e}}_{\mathbf{k}} - \Delta^{\text{eh}}_{\mathbf{k}}(\omega) + i\gamma^{\text{El-Ph}}\Big)\psi^{\text{he}}_{\mathbf{k}}(\omega) \\ +&\Big(1 - f^{\text{e}}_{\mathbf{k}} - f^{\text{h}}_{\mathbf{k}}\Big)\Big(\mathbf{d}^{eh}_{\mathbf{k}} \cdot \mathbf{E}(\omega) + \frac{1}{\mathcal{V}}\sum_{\mathbf{k}'} V_{|\mathbf{k}-\mathbf{k}'|}\psi^{\text{he}}_{\mathbf{k}'}(\omega)\Big) + \frac{1}{\mathcal{V}}\sum_{\mathbf{k}'} V^{\text{eff,eh}}_{\mathbf{k}\mathbf{k}'}(\omega)\psi^{\text{he}}_{\mathbf{k}'}(\omega) = 0\,. \end{aligned} \tag{18}$$

Just as the well-known Bethe-Salpeter equation in screened ladder approximation [34, 35], the SBE on GW-level describe two-particle states in the presence of a dynamically screened carrier-carrier in-

teraction. The single-particle energies $\varepsilon_{\mathbf{k}}^{\text{HF,e/h}}$ contain renormalization effects on a Hartree-Fock level. The second line contains the light-matter coupling term $\mathbf{d} \cdot \mathbf{E}$ of the material's dipoles to the external field as well as a two-body interaction term facilitating excitonic resonances in the optical response. These terms are modified by a Pauli-blocking factor given by the electron and hole occupancies for which we assume Fermi distribution functions. Consistent with the theory of ionization equilibrium, the Fermi functions describe only quasi-free carriers, while we assume that renormalization effects due to excitons can be neglected. As we focus on the microscopic description of effects induced by excited carriers, we include dephasing contributions due to carrier-phonon interaction on a phenomenological level by adding a constant imaginary part $\gamma^{\text{El-Ph}}$ to the quasiparticle energies. The constant is chosen as $\gamma^{\text{El-Ph}} = 30$ meV according to [25]. Following this reference, we additionally include inhomogeneous broadening caused by impurities by convoluting the susceptibility $\chi(\omega)$ with a gaussian with a FWHM of 32 meV.

All many-body effects induced by carrier-carrier interaction beyond the Hartree-Fock level are contained in the correlation terms

$$V_{\mathbf{kk}'}^{\text{eff,eh}}(\omega) = i\hbar \int_{-\infty}^{\infty} \frac{d\omega'}{2\pi} \left\{ \frac{(1 - f_{\mathbf{k}}^{\text{h}} + n_{\text{B}}(\omega'))2i\text{Im}\, W_{|\mathbf{k}-\mathbf{k}'|}^{\text{ret}}(\omega')}{\hbar\omega - \varepsilon_{\mathbf{k}}^{\text{h}} - \varepsilon_{\mathbf{k}'}^{\text{e}} + i\Gamma_{\mathbf{k}}^{\text{h}} + i\Gamma_{\mathbf{k}'}^{\text{e}} - \hbar\omega'} + \frac{(1 - f_{\mathbf{k}}^{\text{e}} + n_{\text{B}}(\omega'))2i\text{Im}\, W_{|\mathbf{k}-\mathbf{k}'|}^{\text{ret}}(\omega')}{\hbar\omega - \varepsilon_{\mathbf{k}}^{\text{e}} - \varepsilon_{\mathbf{k}'}^{\text{h}} + i\Gamma_{\mathbf{k}}^{\text{e}} + i\Gamma_{\mathbf{k}'}^{\text{h}} - \hbar\omega'} \right\}, \tag{19}$$

with the Bose distribution function $n_{\text{B}}(\omega)$ and Fermi distribution function $f_{\mathbf{k}}^{\text{e/h}}$, and

$$\Delta_{\mathbf{k}}^{\text{eh}}(\omega) = \Sigma_{\mathbf{k}}^{\text{MW,ret,e}}(\hbar\omega - \varepsilon_{\mathbf{k}}^{\text{h}} + i\Gamma_{\mathbf{k}}^{\text{h}}) + \Sigma_{\mathbf{k}}^{\text{MW,ret,h}}(\hbar\omega - \varepsilon_{\mathbf{k}}^{\text{e}} + i\Gamma_{\mathbf{k}}^{\text{e}}), \tag{20}$$

where the Montroll-Ward (MW) self-energy

$$\Sigma_{\mathbf{k}}^{\text{MW,ret},\lambda}(\omega) = i\hbar \int_{-\infty}^{\infty} \frac{d\omega'}{2\pi} \frac{1}{\mathcal{V}} \sum_{\mathbf{k}'} \frac{(1 - f^{\lambda}(\omega - \omega') + n_{\text{B}}(\omega'))2i\,\text{Im}\, W_{|\mathbf{k}-\mathbf{k}'|}^{\text{ret}}(\omega')}{\hbar\omega - \varepsilon_{\mathbf{k}'}^{\lambda} + i\Gamma_{\mathbf{k}'}^{\lambda} - \hbar\omega'} \tag{21}$$

is responsible for quasiparticle renormalizations beyond Hartree-Fock according to

$$\varepsilon_{\mathbf{k}}^{\lambda} = \varepsilon_{\mathbf{k}}^{0,\lambda} + \Sigma_{\mathbf{k}}^{\text{HF},\lambda} + \text{Re}\, \Sigma_{\mathbf{k}}^{\text{MW,ret},\lambda}(\omega)\Big|_{\omega=\varepsilon_{\mathbf{k}}^{\lambda}/\hbar}. \tag{22}$$

The corresponding quasiparticle broadening follows from the imaginary part of the MW self-energy

$$\Gamma^{\lambda}_{\mathbf{k}} = -\mathrm{Im}\,\Sigma^{\mathrm{MW,ret},\lambda}_{\mathbf{k}}(\omega)\Big|_{\omega=\varepsilon^{\lambda}_{\mathbf{k}}/\hbar}\,. \tag{23}$$

For the band-structure energies $\varepsilon^{0,\lambda}_{\mathbf{k}}$ in the absence of excitation-induced renormalizations, we apply an effective mass approximation with the effective masses given in the main text following Ref. [6]. The correlation terms (19)-(21) contain the retarded screened potential $W^{\mathrm{ret}}_{|\mathbf{k}-\mathbf{k}'|} = V_{|\mathbf{k}-\mathbf{k}'|}\varepsilon^{-1}_{\mathrm{exc},|\mathbf{k}-\mathbf{k}'|}(\omega)$, where the inverse dielectric function $\varepsilon^{-1}_{\mathrm{exc},\mathbf{q}}(\omega)$ describes screening due to (unbound) excited carriers in random phase approximation. Additionally, $V_{|\mathbf{k}-\mathbf{k}'|}$ is screened by carriers in filled valence band states, which we describe by an effective dielectric constant (see Supplementary Note 2). The SBE (Supplementary Equation (18)) are numerically solved by matrix inversion for each frequency to obtain the frequency-dependent microscopic polarization and thereby the optical susceptibility using Supplementary Equation (17) (Supplementary Figure 11a), from where we calculate the absorption coefficient (Supplementary Figure 11b).

Supplementary References

[1] Elliott, R. Intensity of optical absorption by excitons. *Phys. Rev.* **108**, 1384 (1957).

[2] Haug, H. & Koch, S. W. *Quantum theory of the optical and electronic properties of semiconductors: fifth edition* (World Scientific Publishing Company, 2009).

[3] Klingshirn, C. F. *Semiconductor optics* (Springer Science & Business Media, 2012).

[4] Manser, J. S. & Kamat, P. V. Band filling with free charge carriers in organometal halide perovskites. *Nat. Photon.* **8**, 737–743 (2014).

[5] Davies, C. L. *et al.* Bimolecular recombination in methylammonium lead triiodide perovskite is an inverse absorption process. *Nat. Commun.* **9**, 293 (2018).

[6] Bokdam, M. *et al.* Role of polar phonons in the photo excited state of metal halide perovskites. *Sci. Rep.* **6**, 28618 (2016).

[7] Semkat, D. *et al.* Ionization equilibrium in an excited semiconductor: Mott transition versus Bose-Einstein condensation. *Phys. Rev. B* **80**, 155201 (2009).

[8] Steinhoff, A. *et al.* Exciton fission in monolayer transition metal dichalcogenide semiconductors. *Nat. Commun.* **8**, 1166 (2017).

[9] Kremp, D., Kraeft, W. & Lambert, A. Equation of state and ionization equilibrium for nonideal plasmas. *Physica A* **127**, 72–86 (1984).

[10] Stolz, H. & Zimmermann, R. Correlated pairs and a mass action law in two-component Fermi systems excitons in an electron-hole plasma. *Phys. Status Solidi (b)* **94**, 135–146 (1979).

[11] Schlanges, M., Bonitz, M. & Tschttschjan, A. Plasma phase transition in fluid hydrogen-helium mixtures. *Contrib. Plasm. Phys.* **35**, 109–125 (1995).

[12] Kremp, D., Schlanges, M. & Kraeft, W.-D. *Quantum statistics of nonideal plasmas*, vol. 25 (Springer Science & Business Media, 2006).

[13] Röpke, G. & Der, R. The influence of two-particle states (excitons) on the dielectric function of the electron-hole plasma. *Phys. Stat. Sol. (b)* **92**, 501–510 (1979).

[14] Saha, M. N. On a physical theory of stellar spectra. *Proc. R. Soc. Lond. A* **99**, 135–153 (1921).

[15] D'innocenzo, V. *et al.* Excitons versus free charges in organo-lead tri-halide perovskites. *Nature Commun.* **5**, 3586 (2014).

[16] Saba, M. *et al.* Correlated electron–hole plasma in organometal perovskites. *Nat. Commun.* **5**, 5049 (2014).

[17] Toyozawa, Y. *Optical processes in solids* (Cambridge University Press, 2003).

[18] Price, M. B. *et al.* Hot-carrier cooling and photoinduced refractive index changes in organic–inorganic lead halide perovskites. *Nat. Commun.* **6**, 8420 (2015).

[19] Yang, Y. *et al.* Observation of a hot-phonon bottleneck in lead-iodide perovskites. *Nature Photon.* **10**, 53 (2016).

[20] Kuzmenko, A. B. Kramers–Kronig constrained variational analysis of optical spectra. *Rev. Sci. Instrum.* **76**, 083108 (2005).

[21] Dušan, G. What is the true Kramers-Kronig transform? *Ceramics-Silikáty* **46**, 25–27 (2002).

[22] Jellison Jr, G. *et al.* Characterization of thin-film amorphous semiconductors using spectroscopic ellipsometry. *Thin Solid Films* **377**, 68–73 (2000).

[23] Even, J., Pedesseau, L. & Katan, C. Analysis of multivalley and multibandgap absorption and enhancement of free carriers related to exciton screening in hybrid perovskites. *J. Phys. Chem. C* **118**, 11566–11572 (2014).

[24] Yang, Y. *et al.* Comparison of recombination dynamics in $CH_3NH_3PbBr_3$ and $CH_3NH_3PbI_3$ perovskite films: influence of exciton binding energy. *J. Phys. Chem. Lett.* **6**, 4688–4692 (2015).

[25] Wright, A. D. *et al.* Electron–phonon coupling in hybrid lead halide perovskites. *Nat. Commun.* **7**, 11755 (2016).

[26] Feneberg, M. *et al.* Band gap renormalization and Burstein-Moss effect in silicon-and germanium-doped wurtzite GaN up to 10^{20} cm^{-3}. *Phys. Rev. B* **90**, 075203 (2014).

[27] Haug, H. & Schmitt-Rink, S. Basic Mechanisms of the optical nonlinearities of semiconductors near the band edge. *JOSA B* **2**, 1135–1142 (1985).

[28] Mahan, G. D. Excitons in metals. *Phys. Rev. Lett.* **18**, 448–450 (1967).

[29] Cui, X. *et al.* Transient excitons at metal surfaces. *Nat. Phys.* **10**, 505–509 (2014).

[30] Asnin, V. M., Stepanov, V. I., Zimmermann, R. & Rösler, M. Coulomb resonance at the Fermi level of the electron-hole liquid in germanium. *Solid State Commun.* **47**, 655–657 (1983).

[31] Livescu, G. *et al.* Free carrier and many-body effects in absorption spectra of modulation-doped quantum wells. *IEEE J. Quantum Elect.* **24**, 1677–1689 (1988).

[32] Olbright, G. R. *et al.* CW and femtosecond optical nonlinearities of type-II quantum wells. *Phys. Rev. Lett.* **66**, 1358–1361 (1991).

[33] Grivickas, P., Grivickas, V. & Linnros, J. Excitonic absorption above the Mott transition in Si. *Phys. Rev. Lett.* **91**, 246401 (2003).

[34] Strinati, G. Effects of dynamical screening on resonances at inner-shell thresholds in semiconductors. *Phys. Rev. B* **29**, 5718 (1984).

[35] Bornath, T., Schlanges, M., Hilse, P. & Kremp, D. Nonlinear collisional absorption in dense laser plasmas. *Phys. Rev. E* **64**, 026414 (2001).

[36] Versteegh, M. A. M., van Lange, A. J., Stoof, H. T. C. & Dijkhuis, J. I. Observation of preformed electron-hole Cooper pairs in highly excited ZnO. *Phys. Rev. B* **85**, 195206 (2012).

[37] Keldysh, L. V. & Kopaev, Y. V. Possible instability of semimetallic state toward Coulomb interaction. *Soviet Phys. Solid State, USSR* **6**, 2219 (1965).

[38] Moskalenko, S. A. & Snoke, D. *Bose-Einstein condensation of excitons and biexcitons: and coherent nonlinear optics with excitons* (Cambridge University Press, 2000).

[39] Kondo, T. *et al.* Disentangling Cooper-pair formation above the transition temperature from the pseudogap state in the cuprates. *Nat. Phys.* **7**, 21 (2011).

[40] Wakisaka, Y. *et al.* Photoemission spectroscopy of Ta_2NiSe_5. *J. Super. Nov. Magn.* **25**, 1231–1234 (2012).

[41] Jahnke, F., Kira, M. & Koch, S. Linear and nonlinear optical properties of excitons in semiconductor quantum wells and microcavities. *Zeitschrift für Physik B Condensed Matter* **104**, 559–572 (1997).

[42] Schäfer, W. & Wegener, M. *Semiconductor optics and transport phenomena* (Springer Science & Business Media, 2013).

[43] Manzke, G., Klähn, T. & Henneberger, K. Quantum kinetics and linear optical response of semiconductors. *Phys. Stat. Sol. (c)* 1480–1483 (2003).
